# A semi high-throughput whole blood-based flow cytometry assay to detect and monitor *Bordetella pertussis*-specific Th1, Th2 and Th17 responses

**DOI:** 10.3389/fimmu.2023.1101366

**Published:** 2023-02-06

**Authors:** Véronique Corbière, Eleonora E. Lambert, Marine Rodesch, Jacqueline A. M. van Gaans-van den Brink, Alicja Misiak, Elles Simonetti, Anne Van Praet, Audrey Godefroid, Dimitri A. Diavatopoulos, Cécile A. C. M. van Els, Françoise Mascart, PERISCOPE WP5 Task 7 working group

**Affiliations:** ^1^ Laboratory of Vaccinology and Mucosal Immunity, Université Libre de Bruxelles (U.L.B.), Brussels, Belgium; ^2^ Center for Infectious Disease Control, National Institute for Public Health and the Environment (RIVM), Bilthoven, Netherlands; ^3^ Department of Paediatrics, Cliniques Universitaires de Bruxelles, Hôpital Erasme, Université Libre de Bruxelles (U.L.B.), Brussels, Belgium; ^4^ School of Biochemistry and Immunology, Trinity College Dublin, Dublin, Ireland; ^5^ Laboratory of Medical Immunology, Radboud Institute for Molecular Life Sciences, Radboud University Medical Center, Nijmegen, Netherlands; ^6^ Radboud Center for Infectious Diseases, Radboud University Medical Center, Nijmegen, Netherlands; ^7^ Infectious Diseases & Immunology, Department of Biomolecular Health Sciences, Faculty of Veterinary Medicine, Utrecht University, Utrecht, Netherlands

**Keywords:** *Bordetella pertussis*, vaccine, whole blood assay, Th1/Th2/Th17, intracellular cytokine staining, flow cytometry

## Abstract

**Introduction:**

The characterization of *B. pertussis* (Bp) antigen-specific CD4^+^ T cell cytokine responses should be included in the evaluation of immunogenicity of pertussis vaccines but is often hindered by the lack of standardized robust assays.

**Methods:**

To overcome this limitation, we developed a two-step assay comprising a short-term stimulation of fresh whole blood with Bp antigens and cryopreservation of the stimulated cells, followed later on by batch-wise intracellular cytokine analysis by flow cytometry. Blood samples collected from recently acellular (aP) vaccine boosted subjects with a whole-cell- or aP-primed background was incubated for 24 hrs with Pertussis toxin, Filamentous hemagglutinin or a Bp lysate (400µl per stimulation). Antigen-specific IFN-γ-, IL-4/IL-5/IL-13-, IL-17A/IL-17F- and/or IL-22-producing CD4^+^ T cells were quantified by flow cytometry to reveal Th1, Th2, and Th17-type responses, respectively. The frequencies of IFN-γ-producing CD8^+^ T cells were also analyzed.

**Results:**

We demonstrate high reproducibility of the Bp-specific whole blood intracellular staining assay. The results obtained after cryopreservation of the stimulated and fixed cells were very well correlated to those obtained without cryopreservation, an approach used in our previously published assay. Optimization resulted in high sensitivity thanks to very low non-specific backgrounds, with reliable detection of Bp antigen-specific Th1, Th2 and Th17-type CD4^+^ T cells, in the lowest range frequency of 0.01-0.03%. Bp antigen-specific IFN-γ^+^ CD8^+^ T lymphocytes were also detected. This test is easy to perform, analyse and interpret with the establishment of strict criteria defining Bp antigen responses.

**Discussion:**

Thus, this assay appears as a promising test for evaluation of Bp antigen-specific CD4^+^ T cells induced by current and next generation pertussis vaccines.

## Introduction

Pertussis, a bacterial respiratory disease caused by *Bordetella pertussis* (Bp) (1), remains one of the most uncontrolled vaccine-preventable disease despite high vaccine coverage (2). Therefore, new vaccine strategies are urgently required. Whole-cell pertussis (wP) vaccines, first implemented in the 1940s, were replaced in most industrialized countries in the 1990s-2000s by less reactogenic, acellular pertussis (aP) vaccines containing highly purified Bp antigens adsorbed to aluminum hydroxide as adjuvant. Despite clinical efficacy of aP vaccines against disease (3), pertussis outbreaks have been reported in countries where aP vaccines were implemented (4–6), with increases in pertussis cases in aP-primed adolescents (7). Fast waning of immunity as well as a suboptimal quality of immune responses induced by aP vaccines have been suggested to contribute to pertussis resurgence (8). In addition, while aP vaccines protect against symptomatic disease, baboon studies have demonstrated that they do not prevent asymptomatic carriage and transmission of pertussis (9). In contrast, wP-vaccinated non-human primates cleared the infection more rapidly than control animals (9), stressing the importance of the development of next generation vaccines with detailed characterization of the immune responses they induce in addition to demonstrating their protective efficacy.

While serum antibodies are the most currently used biomarker for new vaccine evaluation, the role of CD4^+^ T lymphocytes in protection against pertussis is now largely accepted (10). First described in mouse models (10, 11), the role of antigen-specific IFN-γ-producing CD4^+^ T lymphocytes (Th1) in protection was further demonstrated in a non-human primate model (12). Whereas Th1 responses are also induced in humans following infection and immunization with wP vaccines, aP vaccines induce a Th2-dominated CD4^+^ T cell response that is characterized by production of IL-4, IL-5 and/or IL-13 (13–17). In addition, the induction and protective role of IL-17-producing CD4^+^ T cells (Th17) have been demonstrated in several animal models after wP vaccination and pertussis infection (18–21). Consequently, not only Th1 and Th2-type but also Th17-type CD4^+^ T cell responses need to be evaluated during vaccine studies. Finally, in addition to Bp-specific CD4^+^ T cells, CD8^+^ T lymphocytes were also reported to participate in the IFN-γ response to Bp antigens (15, 22–25). Even though the significance of Bp-specific CD8^+^ T cell response is to our knowledge still unknown, their characterization may merit attention.

Currently available T cell immunoassays comprise proliferation assays, measurement of released cytokine concentrations by ELISA, enumeration of the cytokine-secreting cells by ELISPOT or detection of cytokine-containing T cells by multiparameter flow cytometry (FC). These assays are most often performed on cryopreserved peripheral blood mononuclear cells (PBMC) with inherent potential artefacts induced by cell isolation and freezing/thawing procedures, which may affect the proportion, phenotype and functions of cells, especially of effector cells (26–29). Moreover, using frozen cells for stimulation may compromise the optimal presentation of proteins by antigen-presenting cells to CD4^+^ T lymphocytes (30). Additionally, some of these techniques require long *in vitro* stimulation times with the antigens (15, 16, 31), potentially inducing further artefacts, and, except for FC, they do not allow a characterization of the cytokine-producing cells at the cellular level.

To overcome these limitations, we recently developed a whole blood assay (32), which includes a short *in vitro* stimulation step of whole blood (WB) with Bp antigens, followed by intracellular cytokine staining (ICS) to assess Th1, Th2 and Th17-type cytokine-producing and activated T lymphocyte populations specific for Bp antigens. This technique was successfully used for the characterization of human antigen-specific CD4^+^ T cell responses in a booster vaccination study (33). However, in a clinical study setting, antigen stimulation of fresh blood samples followed by antibody staining of cells and FC analysis is logistically challenging and does not allow for simultaneous measurements of longitudinal samples from the same individual.

To address these limitations, we introduced a cryopreservation step after stimulation of whole blood with Bp antigens, based on an approach that was successfully developed to characterize mycobacteria-specific CD4^+^ T cell responses (34, 35). We further optimized and validated this assay on fresh blood samples from recently vaccinated subjects to demonstrate feasibility and accuracy to detect and characterize low Bp antigen-specific Th1/Th2/Th17 CD4^+^ T cell responses.

## Material and methods

### Ethical approval

This study was approved by the Ethics Committee ULB-Hôpital Erasme (aggregation number OMO21, study protocol P2018/515), and all the subjects gave their written informed consent.

### Subjects and whole blood sampling

Blood samples were collected from 12 wP-primed adults and two 5-6-years-old aP-primed children after the administration of an aP vaccine booster. Nine adults were recently boosted (between D13 and D37) whereas the three others received the boost some years before the blood sampling, with a maximum of 6 years (Boostrix^®^, GSK Biologicals, Belgium). The two children were recently boosted (between 1-2 months; Tetravac™ -Sanofi Pasteur, Lyon, France). Mean age of the adults was 33 years (21-52 years in the range), whereas the children were 5-6 years-old. Venous blood samples were collected in sodium heparin tubes (BD Biosciences, Erembodegem, Belgium) and transported directly to the laboratory.

### Antigens for *in vitro* stimulation

Pertussis toxin (PT) and filamentous hemagglutinin (FHA), the two Bp antigens present in all aP vaccines (36), were selected for *in vitro* stimulation of blood cells in the Bp-specific whole blood intracellular staining (BpWB-ICS) assay to expand vaccine-induced Bp-specific precursor T lymphocytes. A Bp lysate (BPL) was also used to detect cellular immune responses to Bp antigens that are not included in aP vaccines. A genetically detoxified PT mutant, with an inactivated S1 subunit (R9K, E129A), was chosen for the *in vitro* T cell assay (LIST Biological laboratories Inc., Campbell, CA, USA, #184) to avoid the toxic activity on target cells (37). PT was resuspended following the manufacturer’s instructions and stored in aliquots at 500 µg/mL at 4°C. The endotoxin content determined by a kinetic chromogenic LAL assay was 0.02 EU/µg. Prior to use, each aliquot of PT was heat-inactivated at 80°C during 10 minutes using a Polymerase Chain reaction block, as recommended to abolish *in vitro* mitogenic activity of the B oligomer on T lymphocytes (16, 31, 37). Heat-inactivated aliquots were kept at 4°C until use. FHA was kindly provided by Sanofi Pasteur (Marcy-l’Étoile, France) and stored at 700 µg/mL at 4°C. The endotoxin content was below the detection limit of 0.005 EU/µg FHA. The BPL of strain B1917 (38) was kindly provided by Q Biologicals (Ghent, Belgium) and stored at 3.4 mg/mL at -80°C. A Bp sonicate, initially used for assay optimization before the availability of the BPL, was kindly provided by K. Mills (TCD, Dublin, Ireland) and stored at 596 µg/mL at -80°C. BPL and BP sonicate were heat-inactivated as described above for PT. All Bp antigens were aliquoted in low-binding tubes (Maxymum recovery from Axygen, VWR, Leuven, Belgium) for long-term storage. TT used to assess the intra-assay reproducibility, was purchased from Calbiochem (Sigma-Aldrich, Bornem, Belgium) and stored at 200 µg/mL at -20°C. As a positive control, *Staphylococcus aureus* enterotoxin B (SEB) was purchased from Sigma (Sigma-Aldrich) and stored at 1 mg/mL at -20°C.

### Short-term whole blood stimulation

Blood samples were processed within 3 hrs maximum of their collection (median of 30 min, 25^th^ percentile - 75^th^ percentile (P25-P75): 18-60 min, range: 15-180 min), consistent with other studies that recommended a short delay between blood collection and processing (34, 39). Freshly drawn whole blood samples were diluted 1:1 with RPMI 1640 medium (Invitrogen/ThermoFisher, Merelbeke, Belgium), supplemented with 40 µg/ml gentamycin (Invitrogen/ThermoFisher). Costimulatory anti-CD28 and anti-CD49d antibodies (clones L293 and L25.3, respectively, BD Biosciences, Mountain View, CA) were added to the diluted blood, each at a final concentration of 1 µg/mL to increase cytokine expression in antigen-specific T cells (34). Two-fold diluted blood was distributed in 15 ml round-bottom polypropylene tubes, each of which containing 800 µl of diluted blood (corresponding to 400 µl original blood volume) and incubated in the absence (negative control) or presence of antigen, i.e. 5 µg/mL PT, 5 µg/mL FHA, 10 µg/mL BPL, 10 µg/mL TT, 10 µg/mL Bp sonicate or 1 µg/mL SEB. The optimal antigen concentrations were either based on the literature (BP sonicate, TT) or on preliminary experiments from the PERISCOPE Consortium (PT, FHA, BPL). To assess intra-assay variability, some stimulations were performed in duplicate. Tubes were loosely covered by caps and incubated for 24 hrs at 37°C and 5% CO_2_. During the last 4 to 5 hrs of incubation, 10 µg/ml Brefeldin-A (Sigma-Aldrich) together with 1/1,000 Monensin (BD Biosciences, Erembodegem, Belgium) was added to each tube to inhibit cellular protein transport (26). Cells were subsequently pelleted at 500 x *g* for 10 min.

### Blood cell processing for direct flow cytometry analysis or cryopreservation

The protocol was adapted from the BD protocol (Alternative Protocol, Activation and Intracellular Staining of Whole Blood, BD Biosciences). Briefly, erythrocytes were lysed by adding 10 mL BD PharmLyse (BD Biosciences) to the pelleted cells for 10 min at room temperature (RT) in the dark. After washing with phosphate buffered saline (PBS, Westburg – Lonza, The Netherlands), cells were centrifuged at 500 x *g* for 10 min, the supernatants were discarded, and the white blood cells were fixed by incubation with 1 mL of the Fixation/Permeabilisation solution (from the BD Cytofix/Cytoperm Fixation/Permeabilisation kit, BD biosciences) during 20 min at RT in the dark. After centrifugation at 500 x *g* for 5 min, the white blood cells were cryopreserved in 500 µl Recovery Cell Culture Freezing medium (ThermoFisher), a formulation based on Dulbecco’s Modified Eagle Medium (High Glucose) with optimized levels of foetal bovine serum, bovine serum and 10% DMSO per condition. Cells were transferred into cryovials transferred into cryovial, before storage at -80°C for delayed analysis by batches. In case of direct staining and flow cytometry acquisition, the white blood cells were directly permeabilised by incubation with 2 mL of the Perm/Wash solution (from the BD Cytofix/Cytoperm Fixation/Permeabilisation kit, BD biosciences) for 10 min at RT in the dark.

### Thawing and permeabilization of the stimulated and fixed cells after cryopreservation

Cryovials containing the stimulated, fixed and frozen white blood cells were retrieved from the -80°C freezer and thawed in a water bath at 37°C for 2 min. Thawed cells were transferred from cryovials to 15 mL conical tubes containing 5 mL PBS. After centrifugation at 500 x *g* for 5 min, the cells were permeabilized by adding 2 mL of the Perm/Wash solution (BD Biosciences) and incubated at RT for 10 min.

### Staining of the cells for flow cytometry and acquisition of the data

After permeabilization, either directly on freshly stimulated cells or after cryopreservation of the stimulated and fixed cells, surface and intracellular antibody staining of the cells was combined and performed in the presence of Perm/Wash buffer during 30 min at RT in the dark. Cells were stained with one of the two monoclonal antibody panels; panel 1: CD3-PcP, CD4-APC H7, CD8-PE, CD14/CD19-V500, IFN-γ-BV421, IL-5/IL-13-APC, IL-17A-FITC (Details see **Table 1**, panel 1); or panel 2: CD3-APC-H7, CD4-BB515, CD8-PerCP-Cy5.5, IFN-γ-BV421, IL-4-APC, IL-5-APC, IL-13-APC, IL-17A-PE, IL-17F-PE, IL-22-PE-Cy7 (Details see **Table 1**, panel 2). The antibody panel 1 was optimised in order to improve the detection of Th1 and Th17-type responses. After staining, cells were washed with 2 mL Perm/Wash buffer, transferred in FACS tubes (BD Biosciences), and washed again with PBS before they were recovered in FACS Flow buffer (BD Biosciences) for acquisition.

**Table 1:** Antibody clones and fluorochromes.

	Marker	Clone	Fluorochrome	Company	Reference
Panel 1					
1	IL-17A	BL168	FITC	Biolegend	512304
2	CD8	SK1	PE	BD biosciences	345773
3	CD3	SK7	PcP	BD biosciences	345766
4	IL-5	TRKF5	APC	Biolegend	504305
	IL-13	JES105A2	APC	Biolegend	501908
5	IFN-γ	B27	BV421	BD biosciences	562988
6	CD4	SK3	APC-H7	BD biosciences	641398
7	CD14	M5E2	V500	BD biosciences	561391
	CD19	HIB19	V500	BD biosciences	561121
Panel 2					
1	CD4	SK3	BB515	BD Biosciences	565996
2	IL17A	BL168	PE	Biolegend	512306
	IL17F	033-782	PE	BD Biosciences	561197
3	IL22	22URTI	PECy7	eBiosciences	25-7229-42
4	CD8	SK1	PcP Cy5.5	BD Biosciences	565310
5	IL4	MP-4-25D2	APC	Biolegend	500812
	IL5	TRKF5	APC	Biolegend	504305
	IL13	JES105A2	APC	Biolegend	501908
6	CD3	SK7	APC H7	BD Biosciences	641415
7	IFN-γ	B27	BV421	BD biosciences	562988

Acquisition was initially performed on a FACSCanto II flow cytometer (BD Biosciences) to assess the intra-assay reproducibility and a BD-LSR Fortessa flow cytometer (BD Biosciences) was used subsequently for the next experiments. The content of the tube was completely acquired. Compensations were performed using Comp Beads (BD Bioscience) tubes individually stained with each fluorophore, and compensation matrices were calculated with FACSDiva. Cytometer Setting and Tracking (CST) beads (BD Biosciences) were acquired before each experiment to ensure that cytometer parameters remained consistent across all experiments.

### Flow cytometry analysis

All flow cytometry data analyses were performed with FlowJo software (version 9.5.3, Tree Star, Ashland, OR USA) by using sequential gating. Preliminary experiments using a live/dead dye demonstrated that most cells were alive after the stimulation, and successive gates were applied to exclude dead cells. The gating strategy shown in **Supplementary Figure 1** is an example of cryopreserved cells stained with antibody panel 2. Briefly, after selection of a time of homogenous acquisition, lymphocytes were determined according to their size and granularity. After doublet exclusions and CD3^+^ T cells selection, CD4^+^ CD8^-^ T cells were analysed for their content in cytokines. The numbers of acquired events and percentages of CD4^+^ T cells that produce IFN-γ, IL-4/IL-5/IL-13, IL-17A/IL-17F or IL-22 were reported. The numbers of events recorded in the CD3 gate were 264,500 (median with range: 97,396-533,000) and 344,000 (median with range: 96,432-577,000) for the samples stained freshly and after cryopreservation, respectively. For CD4^+^ T lymphocytes, these numbers were 119,000 (median with range: 43,536-346,000) and 144,500 (median with range: 53,800-377,000), respectively. CD8^+^ T lymphocytes were analysed for their content in IFN-γ, and the numbers of CD8 events were 72,973 (median, range: 17,647-149,000) and 89,034 (median, range: 23,288-164,000), respectively. The numbers of acquired events were thus higher after cryopreservation within the CD3, CD4 and CD8 gates (p=0.0001, p=0.0003, p=0.0004, respectively).

The non-specific backgrounds were defined as the percentage of cytokine-producing CD4^+^ or CD8^+^ T lymphocytes among cells incubated in the absence of antigen. Percentages of antigen-specific cytokine-producing CD4^+^ and IFN-γ-producing CD8^+^ T cells were determined after the non-specific background values were subtracted.

### Criteria defining significant percentages of Bp antigen-specific cytokine-containing cells

To determine whether a Bp antigen-specific T cell response was relevant, we first compared the results obtained for the stimulated conditions to those from unstimulated condition by checking carefully the Flowjo analyses. The percentages of cytokine-containing cells in a diluted whole blood sample after Bp antigen stimulation were arbitrarily defined as significant i.e. different from the non-specific background, based on two criteria: a stimulation index (SI, percentage of cytokine positive cells in Bp antigen-stimulated condition/percentage of cytokine positive cells in the absence of antigen) ≥ 2, and a percentage of Bp antigen-specific cells (i.e. after subtraction of the non-specific background) ≥ 0.030% (**Table 2**). Both a high number of events acquired in the parent gate and a low background in the absence of antigen allowed us to consider low percentages of positive cells in stimulated conditions to be antigen-specific. Results were considered doubtful in two cases (1): a SI > 2 with a percentage of positive cells between 0.010–0.030%, and (2) a SI between 1.5 and 2, with a percentage of positive cells ≥ 0.010%. Results were always considered negative in case of a percentage of positive cells lower than 0.010%. These criteria were validated by checking back the Flowjo analyses.

**Table 2:** Criteria to define responders and doubtful responders to Bp antigens.

	SI	Percentage of positive cells *
**Responder**	≥ 2	≥ 0.030%
**Doubtful responder**	≥ 2 ≥ 1.5 – 2	≥ 0.010% - < 0.030% ≥ 0.010%
**Non-responder**	< 2 or ≥ 2	< 0.010%

SI, Stimulation index (percentage of cytokine positive cells in Bp antigen-stimulated condition/percentage of cytokine positive cells in the absence of antigen); *, non-specific background subtracted.

Subjects with clearly detectable or doubtful percentages of Bp antigen-specific T cells were identified as responders or doubtful responders to that antigen, respectively. Subjects were considered as non-responders when a negative result was recorded.

### Statistical analysis

Graphpad Prism 7.03 for Windows (Graphpad software, La Jolla, CA, USA) was used for statistical analysis. Correlations were evaluated by a non-parametric Spearman test. Wilcoxon matched-pairs signed rank test was applied to compare the results obtained for paired samples. Friedman’s test was used to compared three or more paired groups, with Dunn’s test as *post hoc* analysis to compare two conditions. A value of *p*<0.05 was considered significant. *, *p*<0.05; **, *p*<0.01.

## Results

### Intra-assay reproducibility of the BpWB-ICS assay

**Figure 1:**
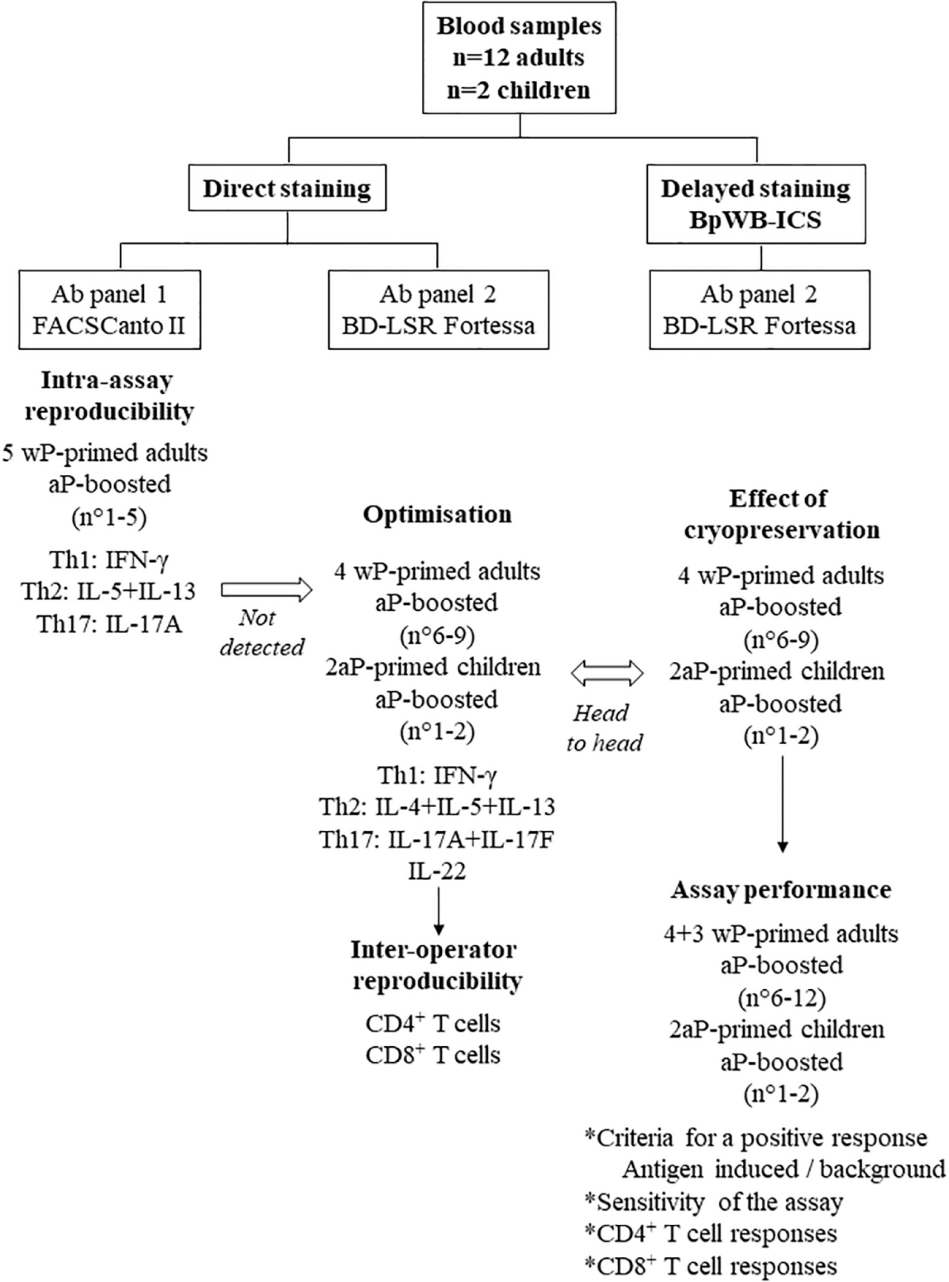
Flow chart of the study with the different steps of development of the BpWB-ICS. Blood samples were collected from 12 adults and from two children. The adults were all primed with a whole-cell pertussis vaccine (wP), whereas the children were primed with an acellular pertussis vaccine (aP). All the subjects were aP boosted. The intra-assay reproducibility was first evaluated on blood samples from five adults, using a direct staining of the Bp-antigen-stimulated cells. As antigen-specific Th2 cells were not detected, further optimisation was performed on blood samples from four adults and from two children. The antibody panel 2 was determined, a BD-LSR Fortessa replaced the FACSCanto II, and the inter-operator reproducibility was evaluated. Results obtained for the same blood samples were analysed head to head without and after a cryopreservation step to develop the BpWB-ICS with a delayed staining of the cells. This allowed us to further analyse the assay performance of the BpWB-ICS.

In order to examine the intra-assay reproducibility of the BpWB-ICS assay, blood samples from five adults (n°1 to n°5) was divided into two aliquots and processed separately from the incubation step until the direct staining of the cells, to detect intracellular cytokines within CD4^+^ T cells (**Figure 1**, **Table 1**, panel 1). The percentages of IFN-γ- or IL-17A-producing CD4^+^ T cells obtained for the duplicates were measured under unstimulated and stimulated conditions with antigens (PT, FHA, Bp lysate, Bp sonicate, TT or SEB).

**Figure 2:**
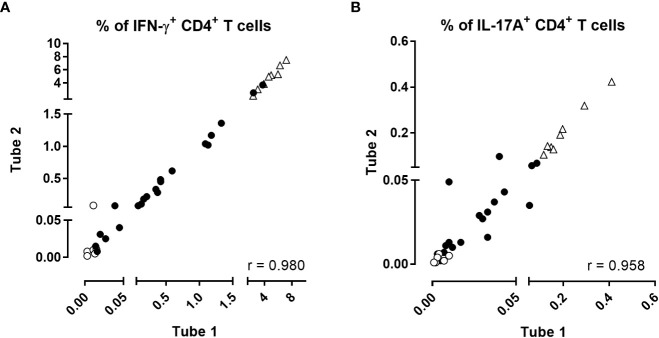
Intra-assay reproducibility of the BpWB-ICS assay. The percentages of **(A)** IFN-γ- and **(B)** IL-17A-producing CD4^+^ T cells obtained after direct staining of two separate aliquots of blood were compared two by two for each stimulation condition. Two different aliquots (400µl/aliquot) from single blood samples were assayed separately from stimulation until the direct staining of the cells. Negative (no antigen, open circles) and positive (SEB, open triangles) controls were used in parallel to blood incubated during 24 hrs with 5 µg/ml PT, 5 µg/ml FHA, 10 µg/ml BPL, 10 µg/ml Bp sonicate or 10 µg/ml TT (black circles). Thirty-five comparisons were performed on five adults (n°1-5), two of which recently aP boosted, two aP boosted less than three years before, and one, six years before. For the two subjects recently boosted, blood was collected before and after aP booster vaccination. After 24 hrs incubation including a 5 hrs protein transport arrest, the cells were fixed, directly permeabilized and stained with antibody panel 1 (**Table 1**). The acquisition was performed on a Canto II flow cytometer, and FlowJo software (version 9.5.3) was used for the analysis. Correlations were evaluated by a non-parametric Spearman test with Graphpad Prism 7.03 software (Graphpad software, La Jolla, CA, USA).

No Th2-containing CD4^+^ T cells were detected whereas the percentages of antigen-induced IFN-γ- or IL-17A-producing CD4^+^ T reached 0.604% and 0.034% in the median, respectively (P25-P75: 0.097%-3.18% for IFN-γ and 0.010%-0.118% for IL17-A). Results obtained in the absence of antigen were very low (0.011% and 0.004% in the median, for IFN-γ and IL-17A, respectively). The results obtained for the two different aliquots of blood were very well correlated (r=0.980, *p*<0.0001 for IFN-γ and r=0.958, *p*<0.0001 for IL-17A (**Figure 2**). The coefficients of variation (CVs) of the results obtained for the duplicates were 16% for IFN-γ and 22% for IL-17A, indicating that the precision of the assay may be considered as good (40, 41).

### Inter-operator reproducibility of data analysis of the BpWB-ICS assay

To assess a potential operator effect on the flow cytometry analyses, we compared results of acquired data obtained for the same samples and analysed with the FlowJo software by two different operators, one experienced and one novice operator.

Blood samples from four adults (n°6 to n°9) and from two children (n°1, n°2), were included here and they were processed with an optimized antibody panel (panel 2, **Table 1**) to further expand the detection of Th2 and Th17-type CD4^+^ T cells (**Figure 1**). As a flow cytometer LSR Fortessa became available, datasets were analysed on this cytometer, and we extent the analysis to CD8^+^ T lymphocytes in addition to CD4^+^ T lymphocytes.

**Figure 3:**
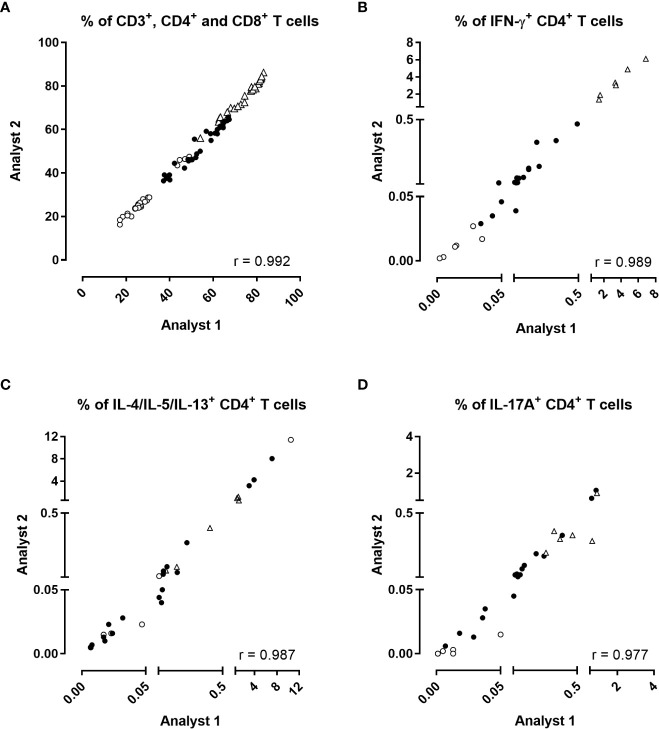
Inter-operator reproducibility of the analysis. The percentages of **(A)** CD3^+^ (open triangles), CD4^+^ (black circles), CD8^+^ (open circles) T cells, and the percentages of **(B)** IFN-γ, **(C)** IL-4/IL-5/IL-13 or **(D)** IL-17A/IL17-F-producing CD4^+^ T cells obtained by two different operators were compared two by two for each stimulation condition after direct staining of the cells. Diluted blood was incubated during 24 hrs with 5 µg/ml PT, 5 µg/ml FHA or 10 µg/ml BPL (black circles). Negative (no antigen, open circles) and positive (SEB, open triangles) controls were used in parallel. Thirty comparisons were performed on samples from four adults (n°6-9), and two 5-6 years-old children, all recently aP vaccine boosted. The cells were fixed, directly permeabilized, and stained with antibody panel 2, optimized for the detection of Th2 and Th17-type responses (**Table 1**). The acquisition was performed on a LSR Fortessa flow cytometer, and FlowJo software (version 9.5.3) was used for the analysis. Correlations were evaluated by a non-parametric Spearman test with Graphpad Prism 7.03 software (Graphpad software, La Jolla, CA, USA).

The percentages of total CD3^+^, CD4^+^ and CD8^+^ T lymphocytes obtained by the two operators were strongly correlated (**Figure 3A**, r=0.992, *p*<0.0001). Similarly, the percentages of IFN-γ-producing-CD4^+^ T cells, IL-4/IL-5/IL-13 and IL-17A/IL-17F were highly correlated (**Figures 3B–D**, *p*<0.0001). The percentages of IL-22-producing CD4^+^ T cells were low but also well correlated **Supplementary Figure 2A**). Similar to CD4^+^ T cells, the percentages of IFN-γ-producing CD8^+^ T cells were also well correlated **Supplementary Figure 2B**). The percentages of other cytokines produced by CD8^+^ T cells were insignificant.

### Effect of cryopreservation in the workflow of the BpWB-ICS assay

To evaluate the effect of cryopreservation of stimulated and fixed cells, blood samples from four adults (n°6 to n°9) and two 5-6 years old-children (n°1, n°2), were divided into two series of aliquots. The first series was stained directly, while the second one was stained after cryopreservation of the stimulated and fixed cells at -80°C during a median of 12 months (range: 1 month – 22 months), for a head to head comparison (**Figure 1**).

**Figure 4:**
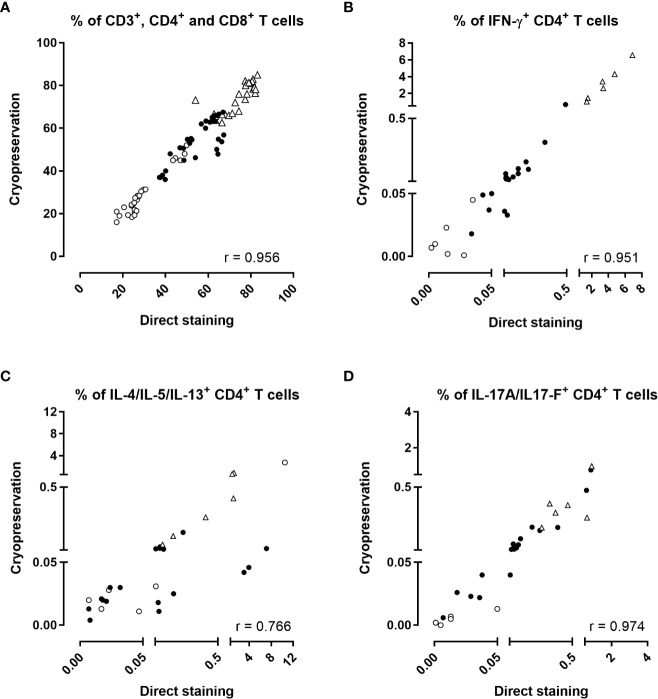
Effect of cryopreservation in the BpWB-ICS assay to detect Bp antigen-specific cytokine-producing CD4^+^ T cells. The percentages of **(A)** CD3^+^ (open triangles), CD4^+^ (black circles), CD8^+^ (open circles), and the percentages of **(B)** IFN-γ, **(C)** IL-4/IL-5/IL-13 or **(D)** IL-17A/IL-17F-producing CD4^+^ T cells obtained by a direct staining procedure were compared to those obtained after cryopreservation of the stimulated and fixed cells. Results were compared two by two for each stimulation condition. Diluted blood was incubated during 24 hrs with 5 µg/ml PT, 5 µg/ml FHA or 10 µg/ml BPL (black circles). Negative (no antigen, open circles) and positive (SEB, open triangles) controls were used in parallel. Thirty comparisons were performed on samples from four adults (n°6-9) and two 5-6 years-old children, all recently aP vaccine boosted. The cells were fixed and then, directly permeabilized for the direct staining, or cryopreserved in the Recovery Freezing Medium (ThermoFisher) for storage at -80°C. Frozen samples were thawed in PBS before permeabilization. The cells were stained with antibody panel 2 (**Table 1**). The acquisition was performed on a LSR Fortessa flow cytometer and FlowJo software (version 9.5.3) was used for the analysis. Correlations were evaluated by a non-parametric Spearman test with Graphpad Prism 7.03 software (Graphpad software, La Jolla, CA, USA).

**Figure 5:**
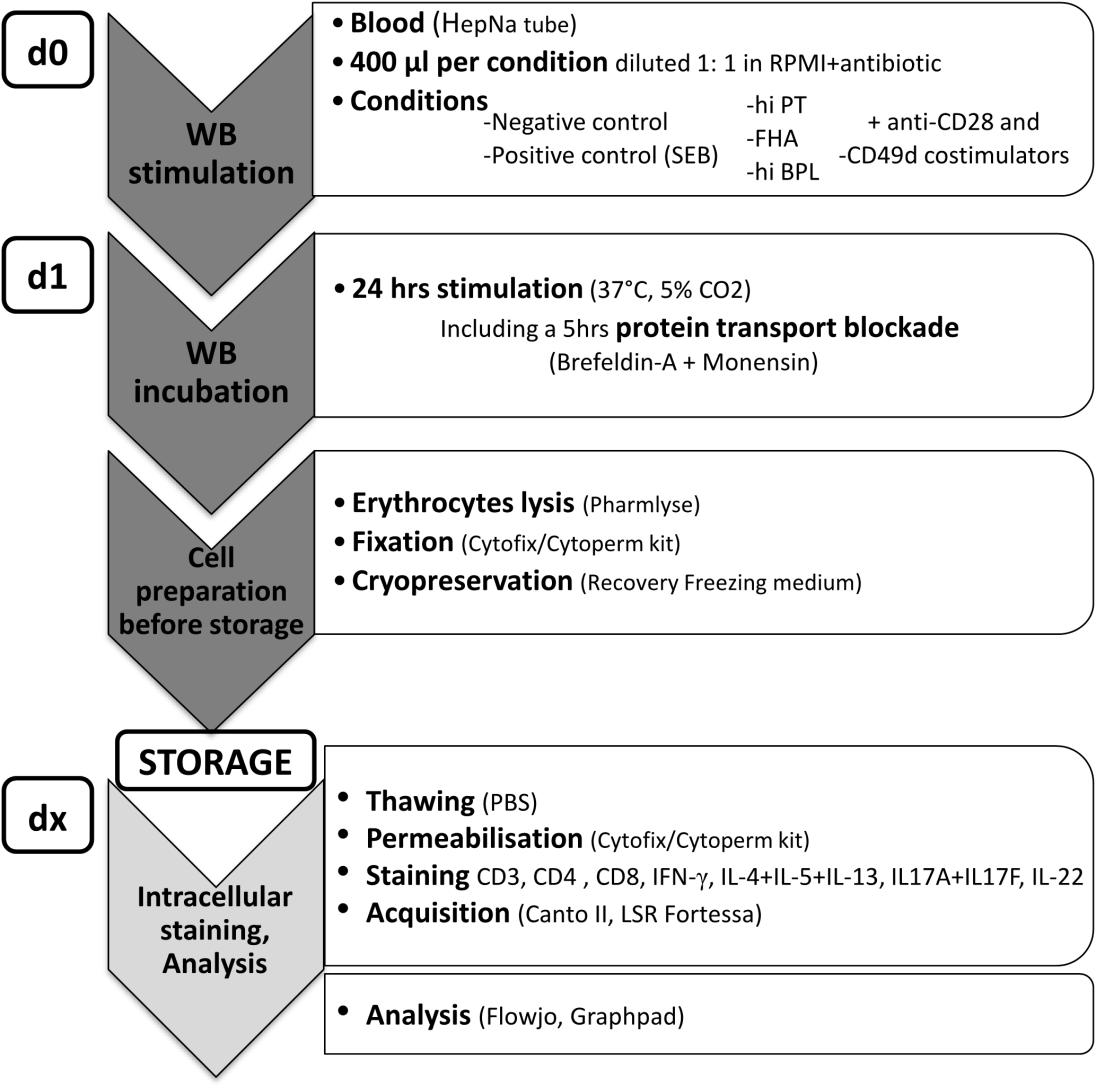
Workflow for the BpWB-ICS assay. Blood was collected in sodium heparin tubes and processed within three hours, maximum. Per stimulation, 400 µl of blood was diluted 1:1 with RPMI supplemented with gentamycin before incubation for 24 hrs in the presence of 1 µg/ml anti-CD28 and anti-CD49 co-stimulants in 15 ml round-bottom polypropylene tube (day 0, d0). Diluted blood was stimulated with 5 µg/ml heat-inactivated (hi) PT, 5 µg/ml FHA or 10 µg/ml hi Bp lysate. Negative (no antigen) and positive (SEB) controls were used in parallel. Protein transport arrest reagents (10 µg/ml Brefeldin-A and 1/1,000 Monensin) were added during the last 5 hrs of the incubation. After 24 hrs incubation (d1), red blood cells were lysed with the Pharmlyse buffer (BD Biosciences), and the white blood cells were fixed with the Fixation/Permeabilisation solution (BD biosciences), before freezing in the Recovery Freezing medium (ThermoFisher) and storage at -80°C for delayed analysis in batches (dx). After thawing in PBS, the cells were permeabilized with the BD Perm/Wash buffer (BD Biosciences), and stained with antibody panel 2 (**Table 1**) for analysis of IFN-γ-, IL-4/IL-5/IL-13-, IL-17A/IL-17F- or IL-22-producing CD4^+^ T cells to reveal Th1, Th2, and Th17-type responses, respectively.

The percentages of total CD3^+^, CD4^+^ and CD8^+^ T lymphocytes obtained from fresh versus frozen samples were highly correlated (**Figure 4A**, r=0.956, *p*<0.0001), as were the percentages of IFN-γ-producing CD4^+^ T cells (**Figure 4B**, r=0.951 and *p*<0.0001). The correlation between the percentages of CD4^+^ T cells-producing IL-4/IL-5/IL-13 obtained by the two procedures was slightly lower albeit still satisfactory (**Figure 4C**, r=0.766, *p*<0.0001). Eight discrepancies out of 30 results were noticed between results of Th2-producing cells obtained by the two procedures, with slightly higher percentages noticed for the direct staining than for the procedure performed after cryopreservation (**Figure 4C**). These discrepancies originated from two adults for several stimulating conditions, and from one child for the unstimulated condition. The percentages of IL-17A/IL-17F-producing CD4^+^ T cells obtained by the two procedures were perfectly correlated (**Figure 4D**, r=0.974 and *p*<0.0001). Good correlations were also found for the very low percentages of IL-22-producing CD4^+^ T cells (r=0.860, *p*<0.0001, **Supplementary Figure 3A**), and for IFN-γ-producing CD8^+^ T cells (r=0.883, *p*<0.0001, **Supplementary Figure 3B**). The non-specific backgrounds were similar for direct staining compared to staining after cryopreservation (**Supplementary Figure 4**). **Figure 5** summarized the final workflow of the BpWB-ICS assay.

### Detection of Bp antigen-specific Th1, Th2 and Th17-type CD4^+^ T lymphocytes by the BpWB-ICS assay

To determine the assay performance and the capacity of the assay to detect Bp antigen-specific Th1, Th2, and Th17-type responses, we compared the percentages of cytokine-producing CD4^+^ T cells of cryopreserved samples stimulated with PT, FHA, BPL or SEB, to the non-specific background, after staining with antibody panel 2 (**Figure 1**). Samples from seven adults (n°6 to n° 12) and from two children (n°1, n°2), all recently aP vaccine boosted, were analysed (**Figure 1**).

Except for Th2-producing cells, background percentages were remarkably low, with median values of 0.012%, 0.007%, and 0.009% for IFN-γ, IL-17A/IL-17F, and IL-22-producing CD4^+^ T cells, respectively (**Supplementary Figure 5**). This background was higher for the percentage of IL-4/IL-5/IL-13-producing CD4^+^ T lymphocytes with a median of 0.031%, although they were still in an acceptable range (**Supplementary Figure 5**).

**Figure 6:**
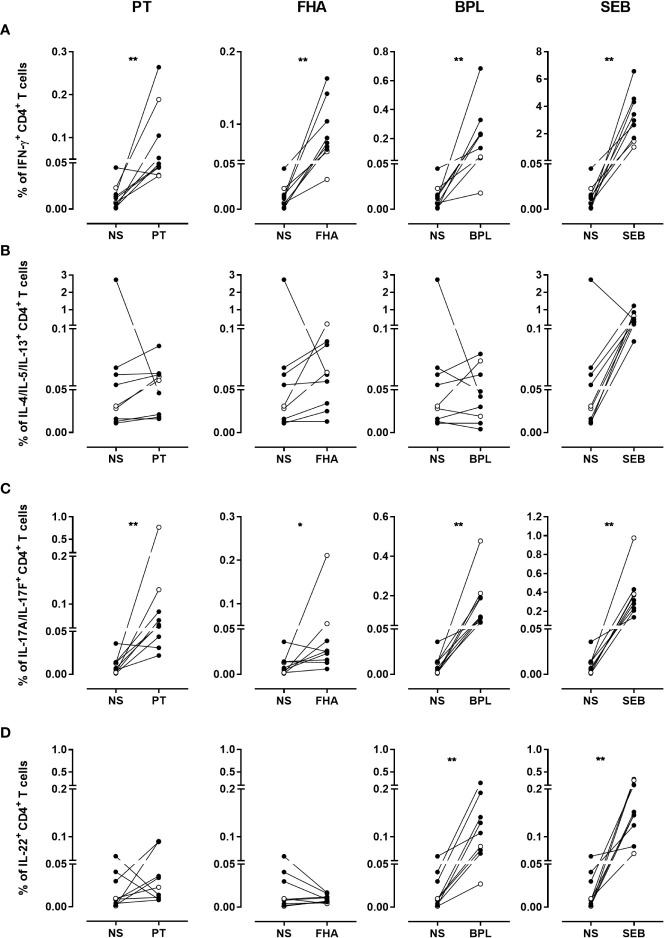
Frequencies of cytokine-producing CD4^+^ T cells in response to PT, FHA, BPL and SEB. The percentages of **(A)** IFN-γ-, **(B)** IL-4/IL-5/IL-13-, **(C)** IL-17A/IL-17F- and **(D)** IL-22- producing CD4^+^ T cells in cryopreserved samples after stimulation with PT, FHA, BPL or SEB are compared to those obtained in the absence of antigen (NS). Diluted blood was incubated during 24 hrs with 5 µg/ml PT, 5 µg/ml FHA or 10µg/ml BPL. Negative and SEB positive controls were used in parallel. The comparison is performed for seven adults (black circles) (n°6-12), and two 5-6 years-old children (open circles), all recently aP vaccine boosted. The cryopreserved cells were processed for intracellular staining as described in **Figure 5**, and stained with antibody panel 2 (**Table 1**). The acquisition was performed on a LSR Fortessa flow cytometer and FlowJo software (version 9.5.3) was used for the analysis. Comparisons were performed by using the Wilcoxon matched-pairs signed rank test with Graphpad Prism 7.03 software. *p<0.05; **p<0.01.

In contrast, the percentages of IFN-γ-producing CD4^+^ T cells obtained after stimulation with PT, FHA, BPL or SEB were higher and significantly different from the background percentages (**Figure 6A**). Overall, the percentages of antigen-induced-IL-4/IL-5/IL-13-producing CD4^+^ T cells were not significantly different from the background percentages, due to one subject with a very high IL-4/IL-5/IL-13 background (**Figure 6B**). Excluding this outlier subject resulted in significant differences between PT, FHA or SEB-stimulated compared to unstimulated cells (*p*=0.016 for PT and FHA and 0.008 for SEB). The percentages of IL-17A/IL-17F-producing CD4^+^ T cells obtained after stimulation were higher and significantly different than the background percentages (**Figure 6C**). Significant percentages of IL-22-producing CD4^+^ T cells, different from the background, were only noticed after stimulation with BPL or SEB (**Figure 6D**).

### Sensitivity of the BpWB-ICS assay to detect CD4^+^ T cell memory in recently aP vaccine boosted subjects

The sensitivity of the assay was assessed after staining cryopreserved samples previously stimulated with PT, FHA, BPL, with antibody panel 2. Samples were from seven adults (n°6 to n°12) and from two children (n°1, n°2), all recently aP vaccine boosted (**Figure 1**).

**Figure 7:**
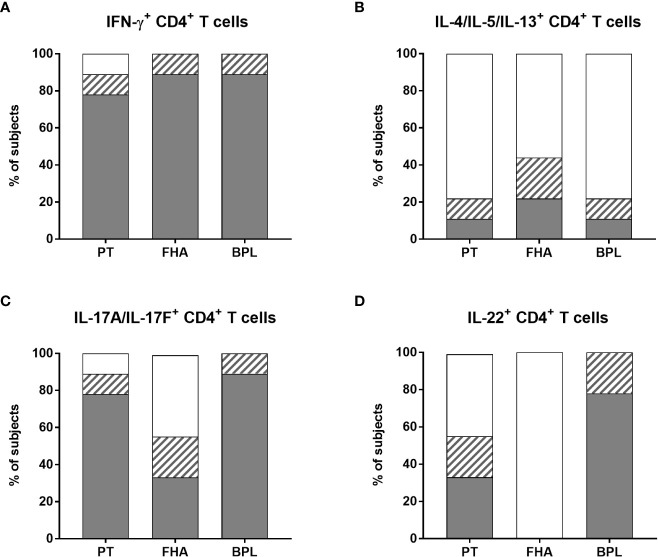
Distribution of non-responders and responders in the BpWB-ICS assay. The percentages of responders (grey areas), doubtful responders (hatched areas) and non-responders (white areas) to PT, FHA and BPL, based on their specific **(A)** IFN-γ-, **(B)** IL-4/IL-5/IL-13-, **(C)** IL-17A/IL-17F-, **(D)** and IL-22-positive CD4^+^ T cells were determined. The analysis was performed for seven adults (n°6-12), and two 5-6 years-old children, all recently aP vaccine boosted. The results obtained after staining of the Bp antigen-stimulated cryopreserved blood cells were considered after subtraction of the non-specific background. The criteria shown in **Table 2** were applied for the classification in responders, doubtful responders and non-responders.

Based on defined criteria of positivity (**Table 2**), PT-specific IFN-γ-producing CD4^+^ T cells were detected for 6/7 adults and 1/2 children (**Figure 7A**), with 0.047% of positive cells (median) and a SI of 8 (median) (**Supplementary Table 1**). The second child had a doubtful response. All the adults (7/7) and one child had FHA- and BPL-specific IFN-γ-producing CD4^+^ T cells, with both high percentages of positive cells and SI (**Supplementary Table 1**). The second child had a doubtful percentage of positive cells (**Figure 7A** and **Supplementary Table 2**). Overall, IFN-γ-producing CD4^+^ T cells were detected to at least one Bp antigen in all subjects.

Bp antigen induced-Th2-type CD4^+^ T cells were clearly detected in the children only. Both children had FHA-specific Th2-type CD4^+^ T cells, one of them had also a PT-specific response, whereas the other one had also a BPL-specific response (**Figure 7B** and **Supplementary Table 1**). The apparent Th2-type CD4^+^ T cell response detected for one adult was non-specific, as it resulted from very high background (**Figure 6**). Some additional Th2-type responses were detected but considered doubtful, and induced by FHA (2/7 adults), PT (the second child), and BPL (1/7 adult) (**Figure 7B** and **supplementary Table 2**).

As for IFN-γ, most subjects had PT-specific IL-17A/IL-17F-producing CD4^+^ T cells (5/7 adults and 2/2 children) (**Figure 7C**), with 0.064% of positive cells (median) and a SI of 41 (median) (**Supplementary Table 1**). Almost all the subjects were responders to BPL (6/7 adults and 2/2 children) (**Figure 7C** and **Supplementary Table 1**). In contrast, only three subjects had detectable FHA-specific IL-17A/IL-17F-producing CD4^+^ T cells (1/7 adults and 2/2 children) (**Figure 7C** and **supplementary Table 1**). In addition, doubtful Th17-type responses were noticed for three adults (one induced by both PT and FHA, a second by FHA, and a third one by BPL) (**Figure 7C** and **Supplementary Table 2**). Of note, even if most subjects had both PT-specific IFN-γ- and IL-17A/IL-17F CD4^+^ T cells, these cytokines were rarely co-expressed by the same cells (**Supplementary Figure 6**), so that Boolean analysis was not further performed.

Finally, PT-specific IL-22-producing CD4^+^ T cells were detected only in three subjects (2/7 adults and 1/2 children), while no FHA-specific IL-22-producing CD4^+^ T cells were noticed (**Figure 7D** and **supplementary Table 1**). In contrast, seven subjects had BPL-induced IL-22-producing CD4^+^ T cells (6/7 adults and 1/2 children) (**Figure 7D** and **supplementary Table 1**). In addition, doubtful IL-22 responses were noticed for four subjects, and induced by PT (1/7 adults and 1/2 children) and BPL (1/7 adults and 1/2 children) (**Figure 7D** and **supplementary Table 2**). Subjects with specific IL-22-producing CD4^+^ T lymphocytes also had IL-17-producing cells, but the cells were different (**Supplementary Figure 6**).

### Detection of Bp antigen-specific IFN-γ-producing CD8^+^ T lymphocytes

The presence of IFN-γ-producing CD8^+^ T cells was investigated in adults n°6 to n°12 and in the two children, following the same procedure as described in **Figure 5**.

**Figure 8:**
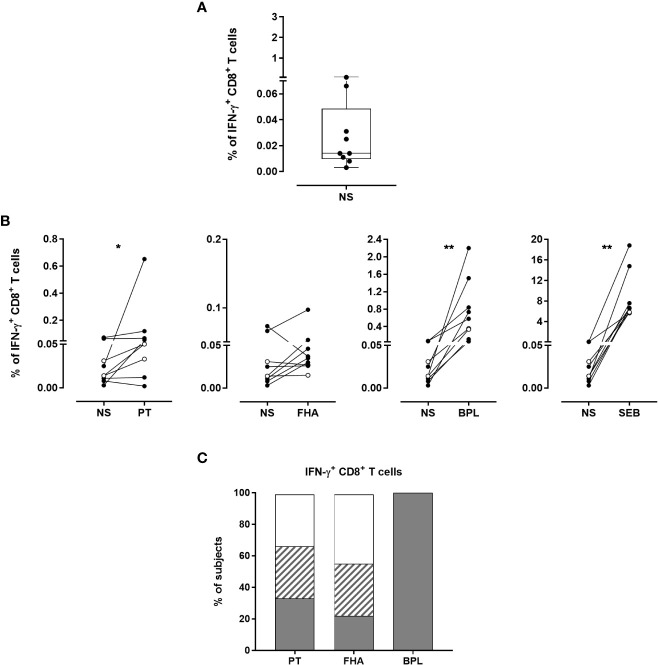
Detection of Bp antigen-specific IFN-γ-producing CD8^+^ T lymphocytes. The presence of IFN-γ-producing CD8^+^ T cells was investigated following the same procedure as described in **Figure 5**. The analysis was performed for seven adults (n°6-12) and two 5-6 years-old children, all recently aP vaccine boosted. **(A)** Non-specific background of IFN-γ-producing CD8^+^ T cells was determined after 24 hrs incubation in the absence of antigen (NS). The cryopreserved cells were processed for intracellular staining as described in **Figure 5**, and were stained with antibody panel 2 (**Table 1**). Horizontal line within the box, box and whisker represent the median, the P25-P75 and the range, respectively. **(B)** The percentages of IFN-γ-producing CD8^+^ T cells obtained after staining of PT-, FHA-, BPL- or SEB-stimulated cryopreserved blood cells were compared to those obtained in absence of antigen (NS). Black and open circles represent the results obtained from adult and children samples, respectively. The Wilcoxon matched-pairs signed rank test with Graphpad Prism 7.03 software was used for statistical analysis. *p<0.05; **p<0.01. **(C)** The percentages of responders (grey areas), doubtful responders (hatched areas) and non-responders (white areas) to PT, FHA and BPL are based on IFN-γ-producing CD8^+^ T cells after background subtraction. The criteria shown in **Table 2** were applied for the classification in responders, doubtful responders and non-responders.

The median background for IFN-γ-producing CD8^+^ T cells was 0.014% (P25-P75:0.010%-0.049%, **Figure 8A**), which was significantly higher than the background of IFN-γ-producing CD4^+^ T lymphocytes (median: 0.012%, P25-P75:0.004%-0.020% (**Supplementary Figure 5**), and *p*=0.039, data not shown). Overall, the percentages of IFN-γ-producing CD8^+^ T cells after stimulation were significantly higher than the background for PT, BPL and SEB, but not for FHA (**Figure 8B**). The same criteria used to determine Bp antigen-specific CD4^+^ T cell responses were applied for IFN-γ-producing CD8^+^ T cells. Even if less frequent than for the CD4^+^ T cells, PT- and FHA-specific IFN-γ-producing CD8^+^ T lymphocytes were detected in 3 and 2 adults, respectively, while BPL induced IFN-γ-producing CD8^+^ T cells in all subjects. In addition, one adult and 2 children had a doubtful response to PT, and three adults to FHA (**Figure 8C**).

## Discussion

The availability of standardized robust assays for the quantification of cytokine-producing CD4^+^ T cells specific for Bp antigens, as described here, is essential for the evaluation of immunological responses to pertussis vaccines. Similar assays, performed on WB, have already been developed and validated in the field of tuberculosis (34, 35) and dengue vaccination study (42), but are, to our knowledge, not yet in use in the field of pertussis vaccines. Previously, we reported on the development of an initial version of a Bp antigen-specific WB FC assay (33). Here we further refined the assay and validated the method for reproducibility. We included a convenience step of cryopreservation of the stimulated cells to enable batch-wise analysis of longitudinal samples from the same individual. This BpWB-ICS assay allows for simultaneous detection of Bp antigen-specific Th1/Th2/Th17-type CD4^+^ and Bp antigen-specific IFN-γ CD8^+^ T cell responses on a total of 2 mL WB, and is thus useful for adult as well as pediatric clinical studies. It is easy to perform, includes negative and positive controls, and is feasible using an eight-color FC.

A main advantage of the BpWB-ICS assay over PBMC ICS assays is that, being performed on WB, it closely reflects the *in vivo* blood immune status, as all cells and components of the blood are present during the stimulation time with antigens (26). PBMC isolation and freezing/thawing procedures that are most often used, require larger blood volumes, are source of pre-analytic variabilities, and have proven difficult to standardize (26, 27). Additionally, such procedures introduce technical bias affecting cellular proportions, phenotypes and functions (26, 27), especially when recall responses are investigated (28, 29), which may lead to potentially biased conclusions (28, 43). In contrast to long *in vitro* stimulation time with antigens, classically used in the pertussis field for the evaluation of lymphocyte proliferation and for measurement of secreted cytokine concentrations (15, 16, 31), we used a short *in vitro* stimulation period, which is more suitable to detect effector memory T cells induced by recent vaccination.

Another major advantage of the BpWB-ICS assay refined here is that staining of cytokine-containing cells and their analysis by FC may be performed later on by batches of selected stored samples, thanks to the addition of a cryopreservation step after stimulation. Here we show that the cryopreservation of the stimulated and fixed cells had no impact on the results, as they were very well correlated to those obtained without cryopreservation. This procedure allows thus for the simultaneous analysis of samples collected at different time points for the same subject in clinical studies, reducing the variability inherent to the cell processing and analysis by FC. It also offers the possibility to select for analysis only samples of interest and for instance to focus only on samples from subjects with a complete longitudinal follow-up. Workflows may thus be easier to organize, and workload can be significantly reduced. In addition, this approach avoids a potential detrimental influence of cryopreservation on antigen presentation of Bp proteins by antigen-presenting cells (30).

We demonstrate here high reproducibility of the results, as well as high sensitivity for detection of rare specific events due to very low non-specific backgrounds. This was obtained thanks to technical improvements resulting in very high numbers of acquired events by FC, to careful optimization of the panel of antibodies used for cell staining, and to the FC analysis strategy as recommended (26, 44). To raise the sensitivity of detection of specific Th2 responses, that are often difficult to evaluate, combined labelling of three different Th2 cytokines (IL-4, IL-5, IL-13) within the same fluorescence channel was performed (15–17, 45). Similarly, labelling for IL-17A and IL-17F was combined to investigate Th17 responses (46). In addition to sensitivity improvement, this approach avoided the need for more than an eight-color FC. Finally, reproducibility of the data analysis *via* FlowJo software by operators with different levels of experience was high. The assay is thus well standardized from the step of blood collection until the FC data analysis provided that the standard operational procedure (**Figure 5**) is strictly applied.

ELISPOT is often reported to be more sensitive than FC to detect antigen-specific T cells among PBMC (47, 48), especially for the detection of low-frequent memory T cells before vaccine booster (49). However, to detect and visualize the cellular IFN-γ recall responses to a protein antigen such as tetanus toxoid after booster vaccine administration, higher sensitivity of FC compared to ELISPOT was demonstrated (49). The superiority of ELISPOT over FC to measure rare events before vaccine booster was suggested to be explained by low cytokine production per memory T cell per time unit, favoring measurement of accumulated cytokines during the *in vitro* culture period (47), as is the case for ELISPOT and not for FC. However, since the BpWB-ICS assay described here was adapted for the detection of very low percentages of positive cells, FC now become the method of choice to evaluate T cell responses induced by pertussis vaccines in clinical studies. Compared to ELISPOT, FC also presents the advantage to be feasible on WB on very small volumes, and to allow for the characterization of the phenotype of the cytokine-producing cells, thereby excluding potential contribution of other cell types, such as NK cells, to cytokine production (50).

This refined version of the BpWB-ICS assay comprises well-defined criteria for positivity of responses. Based on these criteria, we confirmed the presence in blood from recently boosted subjects of Bp-specific Th1-type CD4^+^ T cells, associated with Bp-specific Th2-type CD4^+^ T cells in children who, in contrast to adults, were aP-primed during infancy. In addition, we detected significant Bp antigen-specific Th17 lymphocytes, as most wP-primed adults and the two aP-primed children had PT- and BPL-specific IL-17A/IL-17F-producing CD4^+^ T cells. The induction of IL-17 by pertussis vaccines was only rarely reported in humans until now. They were detected either at very low levels in supernatants of Bp-stimulated PBMC from aP-primed children (51–53), or within CD4^+^ T cells from recently aP boosted individuals with a wP or aP-primed background (33). Considering the contribution of IL-17 in protection against Bp in non-human primates and other animal models (10), optimal detection of IL-17 production, as feasible with the BpWB-ICS assay, is thus a clear added value of this test to be used in human vaccine studies. Interestingly, we observed FHA-specific Th1/Th2/Th17 mixed responses in children, as described in mice immunized with aP vaccines (20). The protective role of Th17 responses against Bp in these cases remains questionable, as Th17 function may be inhibited in a Th2 environment (54, 55). Low percentages of PT-specific IL-22-producing CD4^+^ T cells were also detected, in agreement with previous results in aP boosted subjects (33). The potential role of IL-22 in the defence against *B. pertussis* has to our knowledge not yet been investigated, even if IL-22 has recently attracted great interest being considered as a regulator of host defence in the lung (56).

Finally, the BpWB-ICS assay also detected PT- and FHA-specific IFN-γ-producing CD8^+^ T lymphocytes in most subjects and in response to BPL in all of them. Such Bp antigen-induced cellular immune responses were previously reported during Bp infection and after vaccination in infants and young adults (15, 22–25). Their detection here sustains their involvement in immunity against Bp, but their potential role in protection still requires investigation.

A limitation of the study is that the validation of the refined version of the BpWB-ICS assay was only performed on blood samples from a small number of recently aP vaccine boosted subjects. However, the previous version of the assay was validated by testing blood samples from a large clinical booster vaccination study (33). The major difference of the refined version was the addition of a cryopreservation step and we demonstrated here high correlations of the results obtained head-to-head without and with a cryopreservation step. One might thus reasonably assume that the performance of the refined BpWB-ICS assay will be equally satisfactory in large clinical studies.

In summary, the BpWB-ICS assay described here is an optimal test to characterize both the quantity and the quality of Bp antigen-specific CD4^+^ and CD8^+^ T cells in a very small blood volume, during clinical studies with a longitudinal design. It appears therefore as a promising test to assess the immunogenicity of current and next generation pertussis vaccines.

## Data availability statement

The raw data supporting the conclusions of this article will be made available by the authors, without undue reservation.

## Ethics statement

The studies involving human participants were reviewed and approved by the Ethics Committee ULB-Hôpital Erasme (aggregation number OMO21, study protocol P2018/515). The patients/participants provided their written informed consent to participate in this study.

## PERISCOPE WP5 Task 7 working group

We acknowledge all the participants of WP5 Task 7 within the European PERISCOPE project/consortium:

Prof. Jacques van Dongen (Leiden University Medical Center, Netherlands); CACMvE, JAMvGvdB, EEL (National Institute for Public Health and the Environment RIVM, Netherlands); DAD, ES (Radboud University Medical Center RUMC, Netherlands); Prof. Kingston Mills, AM (Trinity College Dublin, Ireland); Prof. Beate Kampmann, Dr. Thomas Rice (Imperial College of London, United Kingdom); Dr Sophie Roetynck (Medical Research Council-Unit The Gambia at London School of hygiene and tropical medicine, The Gambia); Dr Vanessa Contreras (Commissariat à l'énergie atomique et aux énergies alternatives, INSERM U1184, France); FM, VC (Université Libre de Bruxelles U.L.B., Belgium).

## Author contributions

FM, VC conceived the project and designed the study. VC, MR recruited patients, collected blood samples and clinical data. VC, EEL, JAMvGvdB, AM, ES and DAD participate to the development of the assay. VC optimized the experimental protocol, designed the database, analyzed the data, interpreted the data, and drafted the manuscript. VC, AP, AG performed the experiments for the optimization of the assay. AG analyzed the data. FM interpreted the data, and substantially revised the manuscript. CACMvE, DAD and EEL substantially revised the manuscript. All authors contributed to the article and approved the submitted version.
