# Supplementary material for: A semi high-throughput whole blood-based flow cytometry assay to detect and monitor Bordetella pertussis-specific Th1, Th2 and Th17 responses
Source: Front Immunol. 2023 Feb 6;14:1101366. doi: 10.3389/fimmu.2023.1101366 (PMC9939445; doi:10.3389/fimmu.2023.1101366)
Supplement: Supplementary file 1 [file DataSheet_1.docx]

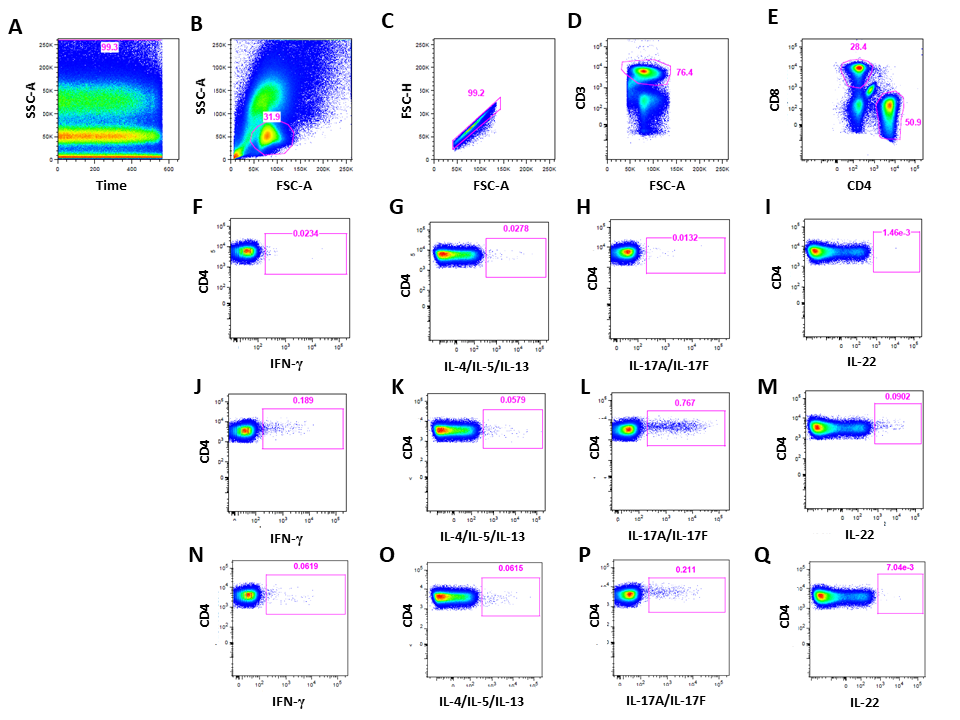


**Supplementary Figure 1.** Gating strategy for FC analysis of the BpWB-ICS assay after cryopreservation of the stimulated and fixed cells. A representative example of the gating strategy for a recently aP vaccine boosted 5-year-old child after antibody staining of cryopreserved cells with panel 2 is shown. After selection of **(A)** a homogenous time of acquisition, **(B)** a gate was drawn in the FSC-A vs SSC-A dot plot to select lymphocytes according to their size and granularity. **(C)** Single cells were identified in a FSC-A vs FSC-H dot plot, before gating on **(D)** CD3^+^ cells in a FSC-A vs CD3 dot plot and subsequently on **(E)** CD4^+^ or CD8^+^ T cells in a CD4 vs CD8 dot plot. Percentages of cytokine positive CD3^+^CD4^+^ T cells without antigen stimulation **(F-I)**, after PT **(J-M)** or FHA stimulation **(N-Q)** were analysed in dot plots of cytokine(s) vs CD4. Rectangular gates represent percentages of total CD4^+^ T cells that produce IFN-γ **(F, J, N)**, IL-4/IL-5/IL-13 **(G, K, O)**, IL-17A/IL17F **(H, L, P)** or IL-22 **(I, M, Q)**. FlowJo software (version 9.5.3) was used for the analysis.


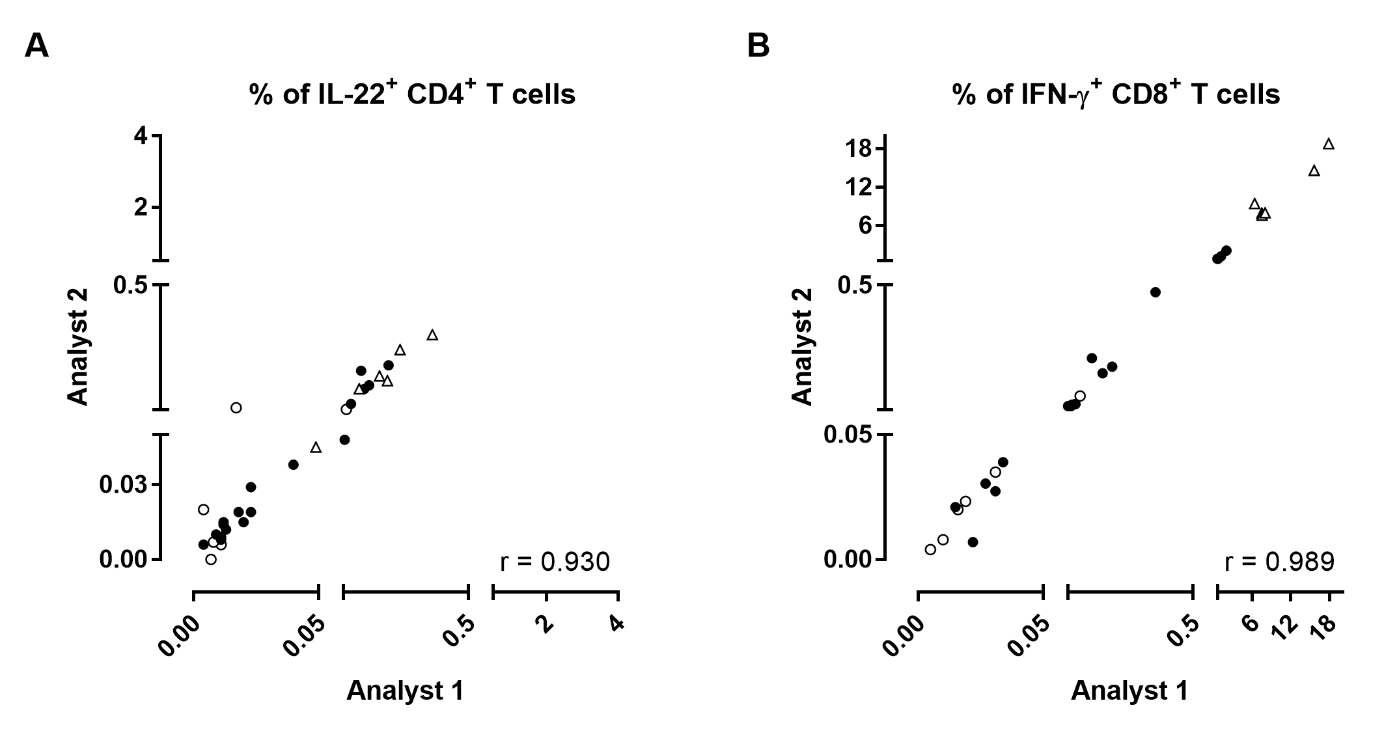


**Supplementary Figure 2.** Inter-operator reproducibility of IL-22-producing CD4^+^ T cells and IFN-γ-producing CD8^+^ T cells analysis. The percentages of **(A)** IL-22-producing CD4^+^ T cells and the percentages of **(B)** IFN-γ-producing CD8^+^ T cells, obtained by two different operators were compared two by two for each stimulation condition after direct staining of the cells. Diluted blood sample was incubated during 24 hrs with 5 µg/ml PT, 5 µg/ml FHA or 10 µg/ml BPL (black circles). Negative (no antigen, open circles) and positive (SEB, open triangles) controls were used in parallel. Thirty comparisons were performed on samples from four adults (n°6-9) and two 5-6 years-old children recently aP vaccine boosted. The cells were fixed, directly permeabilized, and stained with antibody panel 2 (**Table 1**). The acquisition was performed on a LSR Fortessa flow cytometer, and FlowJo software (version 9.5.3) was used for the analysis. Correlations were evaluated by a non-parametric Spearman test with Graphpad Prism 7.03 software (Graphpad software, La Jolla, CA, USA).


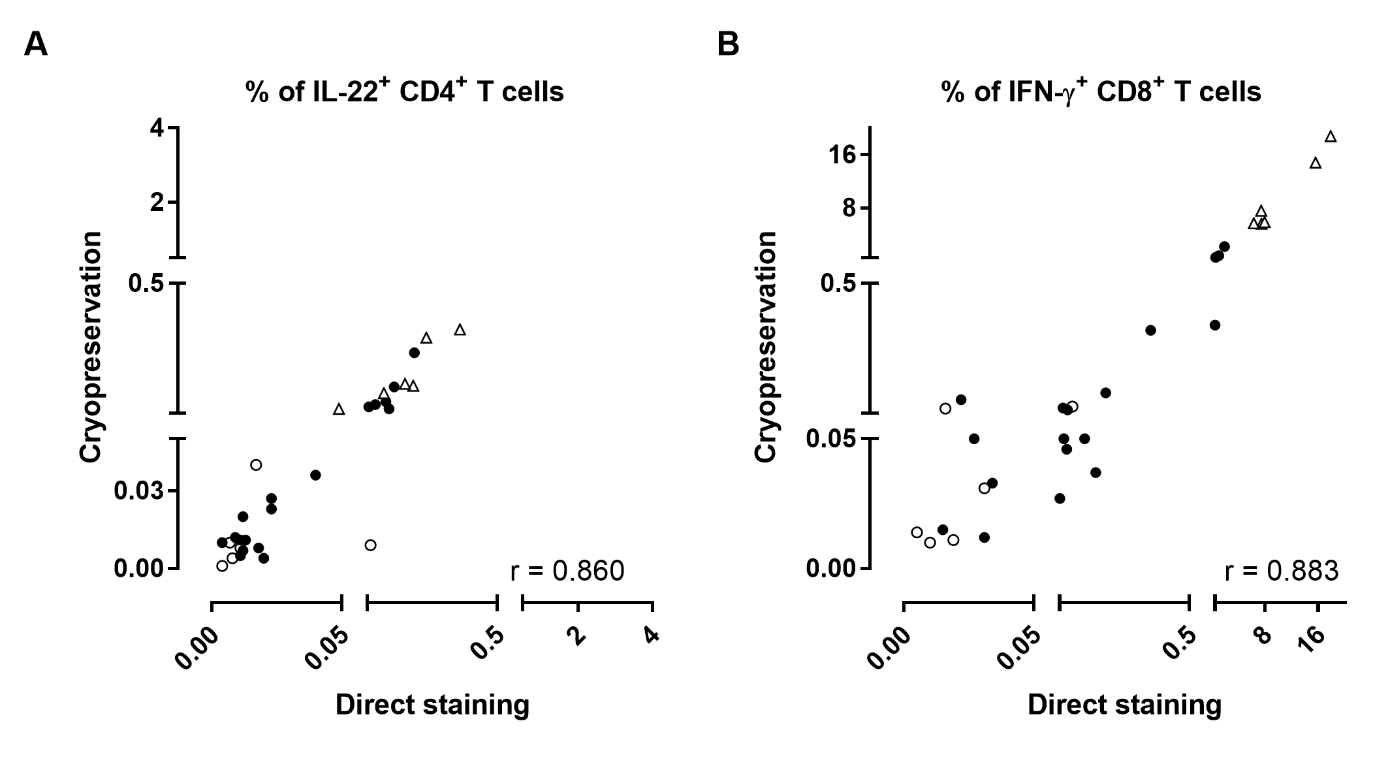


**Supplementary Figure 3.** Effect of cryopreservation in the BpWB-ICS assay to detect Bp antigen-specific IL-22-producing CD4^+^ T cells and IFN-γ-producing CD8^+^ T cells. The percentages of **(A)** IL-22-producing CD4^+^ T cells and the percentages of **(B)** IFN-γ-producing CD8^+^ T cells obtained by a direct staining procedure were compared to those obtained after cryopreservation of the stimulated and fixed cells. Results were compared two by two for each stimulation condition. Diluted blood sample was incubated during 24 hrs with 5 µg/ml PT, 5 µg/ml FHA or 10 µg/ml BPL (black circles). Negative (no antigen, open circles) and positive (SEB, open triangles) controls were used in parallel. Thirty comparisons were performed on samples from four adults (n°6-9) and two 5-6 years-old children recently aP vaccine boosted. The cells were fixed and then, directly permeabilized for the direct staining, or cryopreserved in the Recovery Freezing Medium (ThermoFisher) for storage at -80°C. Frozen samples were thawed in PBS before permeabilization. The cells were stained with antibody panel 2 (**Table 1**). The acquisition was performed on a LSR Fortessa flow cytometer and FlowJo software (version 9.5.3) was used for the analysis. Correlations were evaluated by a non-parametric Spearman test with Graphpad Prism 7.03 software (Graphpad software, La Jolla, CA, USA).


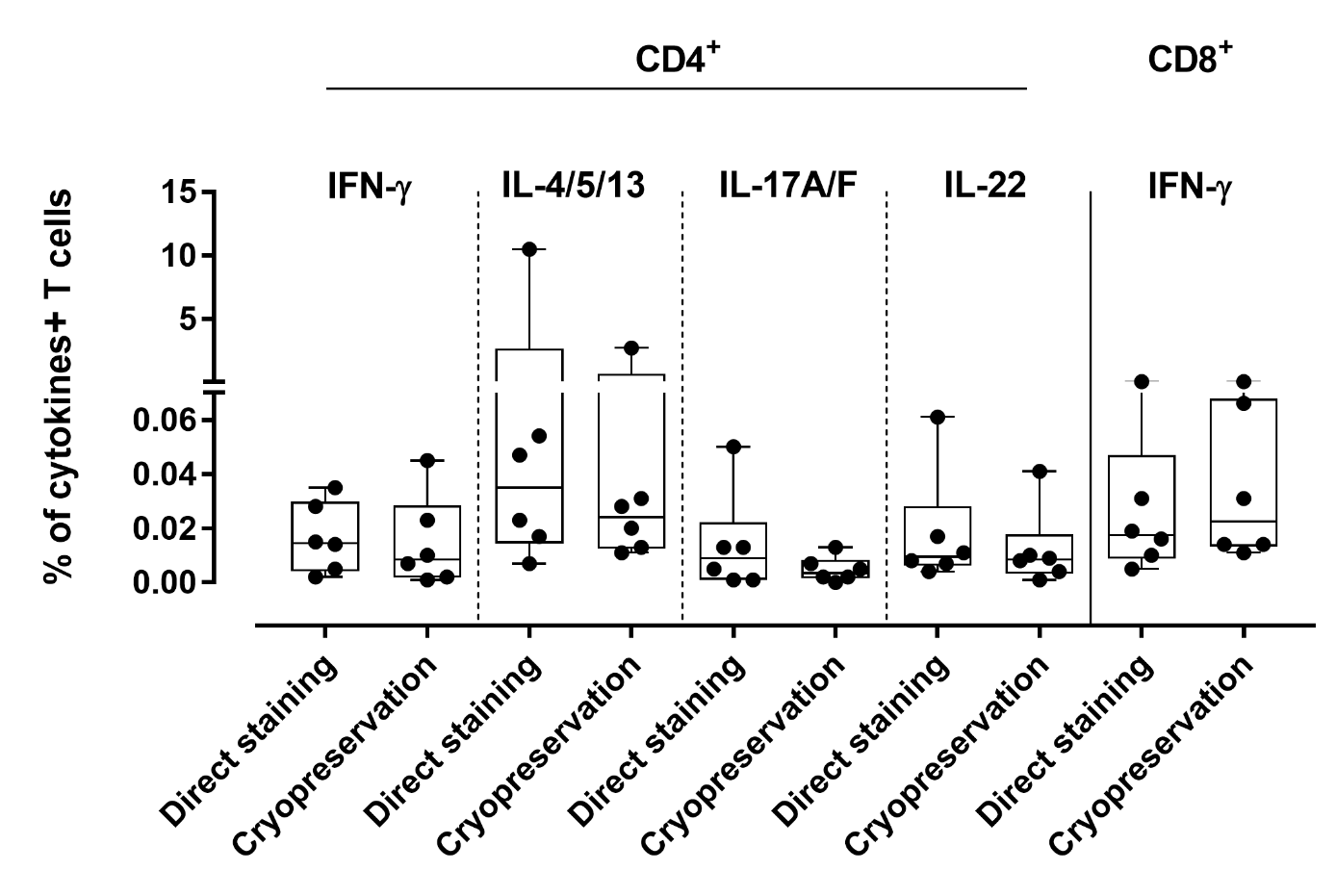


**Supplementary Figure 4.** Effect of cryopreservation on the non-specific background of cytokine-producing CD4^+^ T cells and IFN-γ-producing CD8^+^ T cells. The frequencies of non-specific IFN-γ-, IL-4/IL-5/IL-13-, IL-17A/IL-17F- or IL-22-producing CD4^+^ T cells and the frequencies of non-specific IFN-γ-producing CD8^+^ T cells, detected in the blood samples from four adults (n°6-9) and two 5-6 years-old children recently aP vaccine boosted, in the absence of antigen were determined after 24 hrs incubation. The cells were fixed and then, directly permeabilized for the direct staining, or cryopreserved in the Recovery Freezing Medium (ThermoFisher) for storage at -80°C. Frozen samples were thawed in PBS before permeabilization. The cells were stained with antibody panel 2 (**Table 1**). The acquisition was performed on a LSR Fortessa flow cytometer and FlowJo software (version 9.5.3) was used for the analysis. The data analysis was performed with Graphpad Prism 7.03 software. Horizontal lines within the boxes, boxes and whiskers represent medians, P25-P75 and ranges, respectively. Friedman's test was used to compare the groups, with Dunn’s test as *post hoc* analysis to compare two conditions.


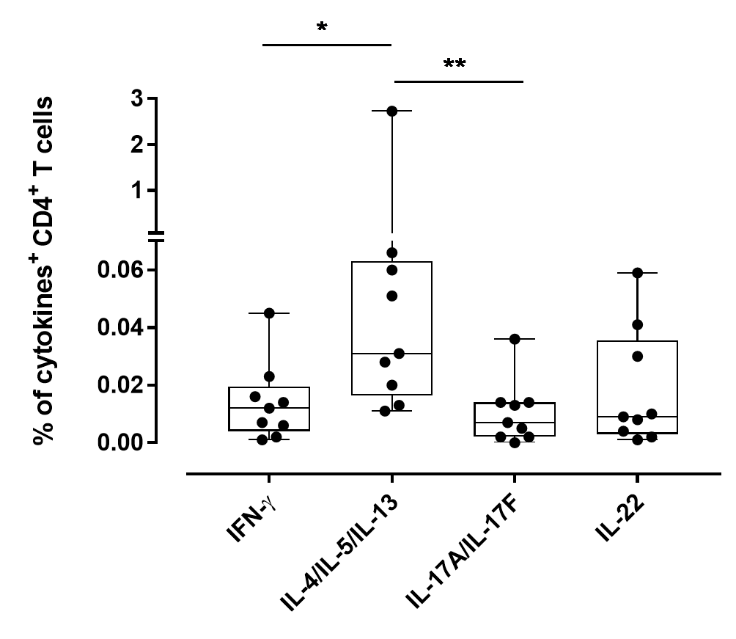


**Supplementary Figure 5.** Non-specific background of cytokine-producing CD4^+^ T cells obtained after cryopreservation of the stimulated and fixed cells. The frequencies of non-specific IFN-γ-, IL-4/IL-5/IL-13-, IL-17A/IL-17F- or IL-22-producing CD4^+^ T cells, detected in the blood samples from seven adults (n°6-12) and two 5-6 years-old children recently aP vaccine boosted, in the absence of antigen were determined after 24 hrs incubation. The cryopreserved cells were processed for intracellular staining as described in Figure 5, and were stained with antibody panel 2 (**Table 1**). The acquisition was performed on a LSR Fortessa flow cytometer and FlowJo software (version 9.5.3) was used for the analysis. The data analysis was performed with Graphpad Prism 7.03 software. Horizontal lines within the boxes, boxes and whiskers represent medians, P25-P75 and ranges, respectively. Friedman's test was used to compare the groups, with Dunn’s test as *post hoc* analysis to compare two conditions. *, *p* <0.05; **, *p*<0.01.


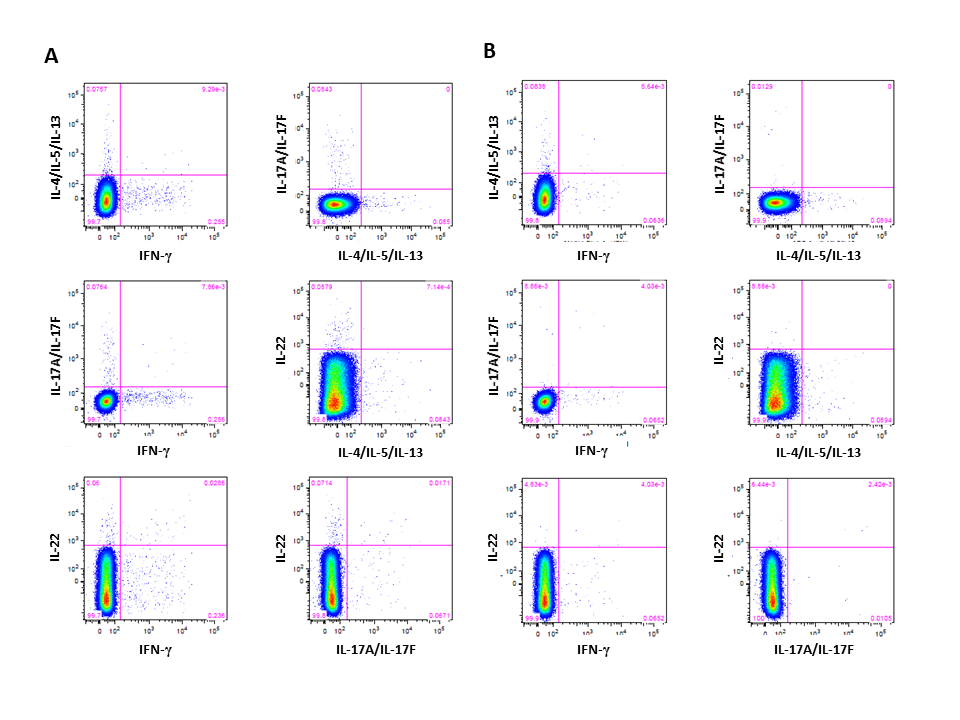


**Supplementary Figure 6.** Co-expression of cytokines by CD4^+^ T cells. A representative example of the analysis for a recently aP vaccine boosted adult after antibody staining of cryopreserved cells with panel 2 is shown. Subsequent to the gating strategy described in Supplementary Figure 1, quadrants with all possible combinations of cytokines were drawn to assess the co-expression of cytokines by CD4^+^ T cells after **(A)** PT stimulation and **(B)** FHA stimulation. FlowJo software (version 9.5.3) was used for the analysis.

**Supplementary Table 1.** Stimulation indexes and percentages of cytokine-producing CD4^+^ T cells in subjects defined as responders based on an unequivocal Bp antigen-specific T cell cytokine response after an aP booster vaccination.

|  |  | **PT** | | **FHA** | | **BPL** | | **SEB** | |
| --- | --- | --- | --- | --- | --- | --- | --- | --- | --- |
|  |  | **SI** | **%*** | **SI** | **%*** | **SI** | **%*** | **SI** | **%*** |
| **IFN-γ** | *N subjects* | *7* | *7* | *8* | *8* | *8* | *8* | *9* | *9* |
|  | Minimum | 3 | 0.032 | 2 | 0.039 | 3 | 0.051 | 59 | 1.013 |
|  | P25 | 4 | 0.034 | 3 | 0.054 | 6 | 0.071 | 93 | 1.552 |
|  | **Median** | **8** | **0.047** | **11** | **0.066** | **26** | **0.217** | **283** | **2.964** |
|  | P75 | 26 | 0.166 | 32 | 0.118 | 52 | 0.301 | 1939 | 4.422 |
|  | Maximum | 131 | 0.248 | 102 | 0.149 | 412 | 0.672 | 5394 | 6.558 |
| **IL-4/IL-5/**  **IL-13** | *N subjects* | *1* | *1* | *2* | *2* | *1* | *1* | *8* | *8* |
|  | Minimum | 2 | 0.030 | 2 | 0.034 | 2 | 0.041 | 5 | 0.076 |
|  | P25 | 2 | 0.030 | 2 | 0.034 | 2 | 0.041 | 7 | 0.154 |
|  | **Median** | **2** | **0.030** | **4** | **0.090** | **2** | **0.041** | **10** | **0.327** |
|  | P75 | 2 | 0.030 | 6 | 0.145 | 2 | 0.041 | 20 | 0.772 |
|  | Maximum | 2 | 0.030 | 6 | 0.145 | 2 | 0.041 | 77 | 1.154 |
| **IL-17A/IL-17F** | *N subjects* | *7* | *7* | *3* | *3* | *8* | *8* | *9* | *9* |
|  | Minimum | 4 | 0.039 | 16 | 0.036 | 7 | 0.059 | 4 | 0.101 |
|  | P25 | 6 | 0.043 | 16 | 0.036 | 12 | 0.082 | 24 | 0.213 |
|  | **Median** | **41** | **0.064** | **25** | **0.052** | **25** | **0.132** | **60** | **0.312** |
|  | P75 | 58 | 0.128 | 35 | 0.198 | 87 | 0.205 | 184 | 0.399 |
|  | Maximum | 59 | 0.754 | 35 | 0.198 | 176 | 0.464 | 232 | 0.963 |
| **IL-22** | *N subjects* | *3* | *3* | *0* | *0* | *7* | *7* | *8* | *8* |
|  | Minimum | 3 | 0.032 |  |  | 6 | 0.061 | 3 | 0.054 |
|  | P25 | 3 | 0.032 |  |  | 6 | 0.063 | 6 | 0.096 |
|  | **Median** | **14** | **0.059** |  |  | **8** | **0.127** | **27** | **0.146** |
|  | P75 | 62 | 0.089 |  |  | 18 | 0.162 | 56 | 0.278 |
|  | Maximum | 62 | 0.089 |  |  | 54 | 0.215 | 214 | 0.333 |

SI: Stimulation index (percentage of cytokine positive cells in Bp antigen-stimulated condition/ percentage of cytokine positive cells in the absence of antigen) ; N: number ; *: percentage of positive cells after subtraction of non-specific background, P25: 25^th^ percentile 25; P75: 75^th^ percentile.

**Supplementary Table 2.** Stimulation indexes and percentages of cytokine-producing CD4^+^ T cells in subjects characterized by a doubtful Bp antigen-specific T cell cytokine response after an aP booster vaccination.

|  | **Subject** |  | **PT** | |  | **FHA** | |  | **BPL** | |  |
| --- | --- | --- | --- | --- | --- | --- | --- | --- | --- | --- | --- |
|  | **number** |  | **SI** | **%*** |  | **SI** | **%*** |  | **SI** | **%*** |  |
| **IFN-γ** | n°2 (child) |  | 5 | 0.029 |  | 5 | 0.026 |  | 3 | 0.011 |  |
| **IL-4/IL-5/IL-13** | n°7 |  | / | / |  | 2 | 0.018 |  | 2 | 0.014 |  |
|  | n°8 |  | / | / |  | 2 | 0.014 |  | / | / |  |
|  | n°2 (child) |  | 2 | 0.025 |  | / | / |  | / | / |  |
| **IL-17A/IL-17F** | n°8 |  | / | / |  | 4 | 0.019 |  | / | / |  |
|  | n°9 |  | 4 | 0.017 |  | 4 | 0.018 |  |  |  |  |
|  | n°10 |  | / | / |  | / | / |  | 2 | 0.032 |  |
| **IL-22** | n°10 |  | / | / |  | / | / |  | 2 | 0.049 |  |
|  | n°6 |  | 5 | 0.028 |  | / | / |  | / | / |  |
|  | n°1 (child) |  | / | / |  | / | / |  | 19 | 0.026 |  |
|  | n°2 (child) |  | 2 | 0.013 |  | **/** | **/** |  | **/** | **/** |  |

SI: Stimulation index (percentage of cytokine positive cells in Bp antigen-stimulated condition/ percentage of cytokine positive cells in the absence of antigen) ; N: number ; *: percentage of positive cells after subtraction of non-specific background.
